# Investigations for diagnosis of secondary hypertension in children: yield and costs

**DOI:** 10.1007/s00467-025-06716-2

**Published:** 2025-03-31

**Authors:** Stefano Guarino, Paola Tirelli, Anna Di Sessa, Giusy Capasso, Federica Auricchio, Luisa De Angelis, Anna Grandone, Emanuele Miraglia del Giudice, Pierluigi Marzuillo

**Affiliations:** Department of Woman, Child and of General and Specialized Surgery, Università Degli Studi Della Campania “Luigi Vanvitelli”, Via Luigi De Crecchio 2, 80138 Naples, Italy

**Keywords:** Hypertension, Costs, Investigations, Children

## Abstract

**Background:**

Screening for secondary hypertension is not recommended for all hypertensive patients, but missing these cases is critical. We aimed to (i) assess hypertension causes in a cohort of hypertensive children, (ii) determine the costs and contributions of an extended diagnostic work-up to screen for secondary hypertension, and (iii) compare the performance of a “short diagnostic work-up” with the protocols of the American Academy of Pediatrics (AAP) and European Society of Hypertension (ESH).

**Methods:**

We conducted a retrospective, single-center study of 70 hypertensive patients aged 1–18 years. All underwent an extended work-up to exclude secondary hypertension. Diagnostic findings, test counts, and costs were analyzed. A short work-up (serum creatinine, fasting glucose, electrolytes, urinalysis, kidney ultrasound (US), and renal artery Doppler US), as well as the AAP and ESH protocols, was evaluated for performance and costs.

**Results:**

Secondary hypertension was identified in 29 patients (41.4%). The extended protocol identified or excluded secondary causes in all patients. Kidney US had the highest diagnostic yield (37.1%). The short work-up and ESH protocol identified all secondary cases, whereas the AAP protocol missed 15 diagnoses. The extended protocol cost € 17,715.60 (€ 253.08 per patient). Direct cost savings were 64.3% with the short work-up, 92.4% with the AAP protocol, and 76.2% with the ESH protocol.

**Conclusions:**

Primary is more common than secondary hypertension in children, with kidney parenchymal disease being the leading secondary cause. As recommended by guidelines, a simplified, focused work-up may offer a cost-effective alternative to extensive screening while maintaining diagnostic accuracy.

**Graphical abstract:**

**Figure:**
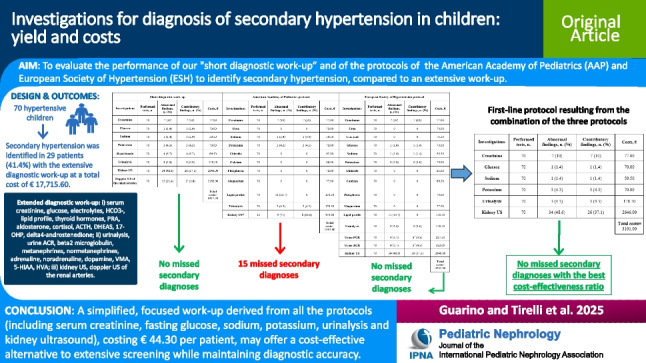
A higher resolution version of the Graphical abstract is available as Supplementary information

**Supplementary Information:**

The online version contains supplementary material available at 10.1007/s00467-025-06716-2.

## Introduction

Valuable guidelines on the management of pediatric hypertension are available [1, 2]. These guidelines recommend a minimal set of tests to be performed for all patients with hypertension, with additional investigations suggested as necessary. However, they lack consistency in specifying which patients should undergo specific screening for secondary causes of hypertension [1, 2]. They generally recommend selecting patients for screening based on factors such as age, medical history, clinical presentation, or difficulty in managing hypertension [1, 2]. Flynn et al. [1] highlighted the need for further evidence, particularly regarding the most effective diagnostic evaluation to confidently exclude secondary causes.

Despite these recommendations [1, 2], in our daily clinical practice, we subjected all pediatric patients diagnosed with hypertension to a comprehensive screening to rule out all potential secondary causes. However, we observed that only a portion of the tests performed contributed meaningfully to diagnosing underlying causes. Therefore, in line with the available guidelines [1, 2], we had the sense that a more streamlined focused screening protocol could efficiently identify the secondary causes of hypertension in children.

For these reasons, we conducted this retrospective study with the aim of assessing the underlying causes of hypertension in a cohort of hypertensive children, the costs associated with our extended diagnostic work-up for ruling out secondary hypertension, and the contribution of these tests to establishing a diagnosis. Additionally, we aimed to evaluate the effectiveness of a “short diagnostic work-up” in identifying patients requiring further investigations for suspected secondary hypertension. Finally, we compared the performance of our short diagnostic work-up with the testing protocols recommended by current guidelines from the American Academy of Pediatrics (AAP) and the European Society of Hypertension (ESH) [1, 2].

## Methods

We retrospectively enrolled all patients aged 1–18 years who consecutively attended our department with suspected hypertension between 2017 and 2024, based on blood pressure measurements conducted either for screening purposes or due to symptoms suggestive of hypertension. Since this study utilized pre-existing, de-identified data, the Institutional Review Board deemed it exempt from approval. According to Italian law, the Authorization to Process Personal Data for Scientific Research Purposes (Authorization No. 9/2014) specifies that retrospective studies using coded data, which cannot be directly traced back to the data subject, do not require ethics approval [3].

As per our clinical practice, we adhered to the guidelines of the American Academy of Pediatrics (AAP) for diagnosing and defining hypertension [1]. Inclusion criteria were (i) age < 18 years; (ii) diagnosis of hypertension or masked hypertension (MH) based on ambulatory blood pressure monitoring (ABPM) for patients ≥ 6 years [4, 5] or diagnosis of hypertension based on office blood pressure measurements for patients < 6 years [6]; and (iii) completion of our comprehensive screening for secondary causes of hypertension (details provided below).

Patients with missing data or a diagnosis of white coat hypertension or elevated blood pressure (previously known as pre-hypertension) were excluded from the study (Supplementary Fig. 1) [1, 4–6]. Additionally, patients with pre-existing chronic kidney disease or a history of kidney transplant were excluded.

### Extended diagnostic work-up

In line with our clinical practice, all enrolled patients underwent a comprehensive first-line diagnostic work-up to exclude potential causes of secondary hypertension. This included (i) on blood sample: measurement of serum creatinine, fasting glucose, electrolytes, bicarbonates, lipid profile, thyroid hormones, plasma renin activity, aldosterone, cortisol, adrenocorticotropic hormone (ACTH), dehydroepiandrosterone sulfate (DHEAS), 17-OH progesterone (17-OHP), delta4-androstenedione; (ii) on urine sample: urinalysis and measurement of creatinine, albuminuria, beta2 microglobulin, metanephrines, normetanephrines, adrenaline, noradrenaline, dopamine, vanillylmandelic acid (VMA), hydroxy-indoleacetic acid (5-HIAA), homovanillic acid (HVA); (iii) instrumental evaluations: kidney ultrasound (US), Doppler US of the renal arteries. If any test produced an abnormal result, it was repeated within 1 month.

Second-line investigations were conducted in cases of abnormalities detected in the renal artery Doppler US. These included computed tomography (CT) angiography or magnetic resonance (MRI) angiography. Additionally, low-dose ACTH stimulation test and dexamethasone suppression test (DST) were performed when clinically indicated.

### Biochemical measurements

Blood samples were collected at 8:00 AM after an overnight fast, with the patient seated for 1 h prior to venipuncture. For toilet-trained patients, 24-h urine samples were collected, whereas for non-toilet-trained patients, urine was collected using a sterile bag. All urinary results were normalized to urinary creatinine levels.

Serum creatinine, fasting glucose, electrolytes, lipid profile, urinary creatinine, albuminuria, and beta2 microglobulin were analyzed through automated clinical chemistry analyzers (Abbott Architect System, Abbott Laboratories, IL, USA). Thyroid hormones were measured by highly specific solid-phase technique–chemiluminescence immunoassays (PerkinElmer, Turku, Finland). Bicarbonates were determined using the Stat Profile Prime analyzer (Nova Biomedical Corporation, Waltham, MA, USA). Aldosterone was measured by radioimmunoassay technique using the Spac-S Aldosterone Kit (Fujirebio, Inc., Tokyo, Japan). Plasma renin activity was quantified by radioimmunoassay technique using the renin activity “FR” kit (Fujirebio, Inc., Tokyo, Japan). ACTH was measured via chemiluminescence immunoassay (Immunodiagnostic Systems, Boldon, UK). Radioimmunological assays were used for the measurement of 17-OHP (OHP-CT: Cis Bio, Gif-sur-Yvette, France), cortisol (Cis Bio, Gif-sur-Yvette, France), delta-4 androstenedione (DRG Diagnostics, Marburg, Germany), and DHEAS (DiaSorin, Saluggia, Italy). For metanephrines, normetanephrines, adrenaline, noradrenaline, dopamine, VMA, 5-HIAA, and HVA, the method was high-performance liquid chromatography (HPLC), using 1260 Infinity II HPLC System (Agilent, Santa Clara, CA, USA).

### Short diagnostic work‑up

To address the potential causes of secondary hypertension, we developed a short protocol a priori, which we then retrospectively tested in our study population. This proposed short protocol includes the following first-line investigations: serum creatinine, fasting glucose, sodium, potassium, bicarbonates, urinalysis, kidney US and Doppler US of the renal arteries.

### Diagnostic work-up proposed by the American Academy of Pediatrics

The latest AAP guidelines recommend that all hypertensive patients should undergo screening including serum creatinine, urea, sodium, potassium, chloride, calcium, phosphorus, magnesium, lipid profile, urinalysis, and kidney US if under 6 years old or if urinalysis or kidney function is abnormal [1].

### Diagnostic work-up proposed by the European Society of Hypertension

According to ESH guidelines, all children with hypertension should undergo routine laboratory tests and imaging studies. These include measurements of serum creatinine, urea, uric acid, glucose, sodium, potassium, chloride, calcium, phosphorus, magnesium, lipid profile, urinalysis, urine protein-to-creatinine ratio (PCR), urine albumin-to-creatinine ratio (ACR), and kidney US [2].

### Data analysis

The number of completed investigations, their associated costs, and diagnostic findings were evaluated. Diagnostic findings were determined through a retrospective review of the underlying etiology of hypertension. The percentage of tests contributing to the final diagnosis was calculated relative to the total number of tests performed.

### Cost analysis

The direct costs for the screening of the 70 enrolled patients were calculated. We used the latest tariff decree issued by the Italian Ministry of Health to estimate these costs as follows [7]: creatinine measurement (€ 1.10), urea (€ 1.00), uric acid (€ 1.05), fasting blood glucose (€ 1.00), sodium (€ 0.85), potassium (€ 1.00), chloride (€ 0.90), calcium (€ 0.95), phosphorus (€ 1.00), magnesium (€ 1.10), total cholesterol (€ 1.05), triglycerides (€ 1.10), bicarbonate (€ 0.95), thyroid-stimulating hormone (TSH) (€ 2.55), free thyroxine (FT4) (€ 2.60), plasma renin activity (€ 6.15), aldosterone (€ 7.80), cortisol (€ 4.45), ACTH (€ 7.20), DHEAS (€ 6.40), 17-OHP (€ 7.70), delta4-androstenedione (€ 6.80), urinalysis (€ 2.55), urinary creatinine (€ 1.90), urinary proteins (€ 3.25), albuminuria (€ 2.70), beta2 microglobulin (€ 3.25), urine metanephrines and normetanephrines (€ 19.80), adrenaline and noradrenaline (€ 19.55), dopamine (€ 15.58), VMA (€ 18.55), 5-HIAA (€ 13.25), HVA (€ 13.55); kidney US (€ 37.80), Doppler US of the renal arteries (€ 45.00), CT angiography (€ 141.45), MRI angiography (€ 184.80), low-dose ACTH stimulation test (€ 16.90), and DST (€ 16.90).

### Evaluation of the target organ damage

Participants underwent echocardiography to measure left ventricular (LV) mass. Left ventricular hypertrophy (LVH) is defined as an LV mass > 51 g/m^2.7^ for both sexes or LV mass > 115 g per body surface area for boys and > 95 g per body surface area for girls [1, 2, 8].

Hypertensive retinopathy was classified using the Wong and Mitchell classification system [9]. Specifically, it was categorized as mild in case of presence of one or more signs among generalized arteriolar narrowing, focal arteriolar narrowing, arteriovenous nicking, and arteriolar wall opacity. It was classified as moderate in case of presence of one or more signs among retinal hemorrhage (blot-, dot-, or flame-shaped), microaneurysm, cotton wool spot, and hard exudates. Finally, it was classified as severe in the case of moderated retinopathy plus optic disc swelling.

### Statistical analysis

Continuous variables were analyzed using an independent-sample *t*-test in cases of normally distributed data and the Mann–Whitney test for non-normally distributed data. Qualitative variables were compared by the chi-square or Fisher exact tests, as appropriate. The Stat-Graph XVII software for Windows (Statgraphics Technologies Inc., VA, USA) was used for all statistical analyses.

## Results

### Study population

During the study period, 115 children were evaluated for suspected hypertension. Of these, 21 were excluded due to the absence of hypertension, 13 for a diagnosis of elevated blood pressure, 6 for white coat hypertension, and 5 due to missing data (Supplementary Fig. 1). Ultimately, 70 patients were enrolled (67.1% male). Among them, 67 had hypertension, and 3 had masked hypertension (Supplementary Fig. 1). Table 1 summarizes the general characteristics of the enrolled patients. The age distribution and causes of hypertension by age are presented in Supplementary Fig. 2. Two patients (2.8%) were younger than 5 years, 20 patients (28.7%) were between 5 and 10 years old, and 48 patients (68.5%) were between 11 and 17 years old. The prevalence of secondary hypertension was 100% (2 out of 2 patients) in those younger than 5 years, 50% (10 out of 20 patients) in the 5–10-year age group, and 35.4% (17 out of 48 patients) in those older than 10 years (Supplementary Fig. 3).

**Table 1:** Clinical characteristics of the 70 enrolled patients

Age, yr, mean (SDS)	12.8 ± 4.7
Male gender, No. (%)	47 (67.1)
Birth weight, kg, mean (SDS)	3.03 ± 0.63
Birth weight < 10th percentile, No. (%)	5 (7.1)
Preterm birth, No. (%)	12 (17.1)
Gestational age, weeks, median (IQR)	37.9 ± 1.8
Symptoms of HTN, No. (%)	30 (42.9)
Obesity, No. (%)	34 (48.6)
Overweight, No. (%)	14 (20)
Left ventricular hypertrophy, No. (%)	4 (5.7)
Hypertensive retinopathy, No. (%)	5 (7.1)

For normally distributed variables, means ± SDS are shown, while for non-parametric ones, median and interquartile range are shown

*HTN,* hypertension; *IQR*, interquartile range; *SDS*, standard deviation score

### Clinical manifestations

Among the 70 enrolled children, 30 (42.9%) presented with symptoms, the most common being headache, often accompanied by flushing and dizziness (Supplementary Fig. 4). Of the 30 symptomatic patients, only 6 (20%) were found to have a secondary cause of hypertension, compared to 23 (57.5%) of the 40 asymptomatic patients (*p* = 0.003). Four patients presented with LVH, and five had hypertensive retinopathy. The mean age at diagnosis of hypertension was 12.8 ± 4.7 years.

### Causes of hypertension

**Fig. 1:**
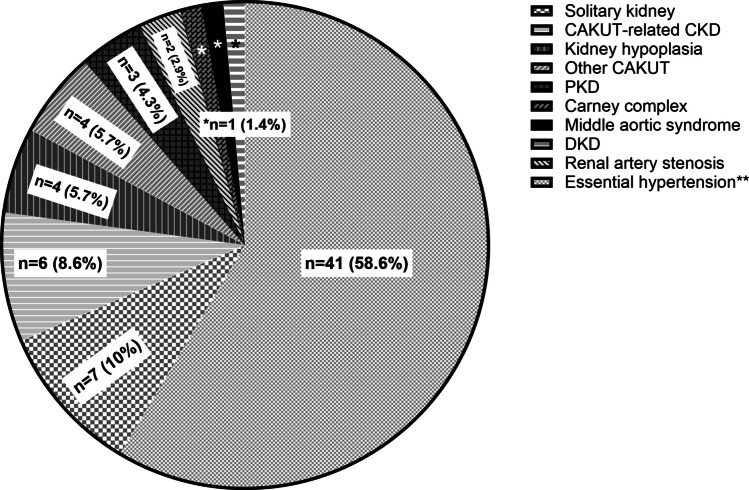
Causes and risk factors for hypertension. Each segment of the chart indicates the absolute number and percentage of patients with the respective cause or risk factor for hypertension. **Essential hypertension (*n* = 41, 58.6%): 27 patients with only obesity and 14 with no identified cause or risk factor. PKD, polycystic kidney disease; CAKUT, congenital anomalies of the kidney and urinary tract; CKD, chronic kidney disease; DKD, diabetic kidney disease

The underlying causes of hypertension are detailed in Fig. 1. The most common were congenital solitary kidney, congenital anomalies of the kidney and urinary tract (CAKUT)-related chronic kidney disease (CKD), kidney hypoplasia, other CAKUT, polycystic kidney disease (PKD), renal artery stenosis, diabetic kidney disease (DKD), Carney complex, and middle aortic syndrome in a patient with Williams syndrome (Fig. 1).

Overall, secondary hypertension was identified in 29 of the 70 children (41.4%). Among them, two patients (one patient with Williams syndrome and one with type 1 diabetes) were diagnosed during follow-up for their specific condition and underwent the extended diagnostic protocol to rule out other potential causes of hypertension. The extended protocol of tests was useful in defining the cause of hypertension (solitary kidney, CKD, kidney hypoplasia, other CAKUT, PKD, renal artery stenosis, Carney complex) in a total of 27 out of 70 patients.

Additionally, 34 children (48.6%) presented with obesity; 27 were solely obese, while 7 had obesity along with a secondary cause of hypertension. No cause or risk factor was identified in 14 patients (20%) (Fig. 1).

### Treatment

All 70 patients were initially recommended non-pharmacological therapy as per guidelines [1], focusing on lifestyle interventions such as the DASH diet (Dietary Approaches to Stop Hypertension), physical activity, and stress reduction [10]. Non-pharmacological therapy alone was sufficient to reduce blood pressure in 14 patients (20%). The remaining 56 patients required pharmacological treatment (54 with hypertension and 2 with masked hypertension). In cases of secondary hypertension, medication was administered until the underlying cause was resolved.

Among the 56 treated patients, 19 (33.9%) required more than one anti-hypertensive drug to achieve optimal blood pressure control. In 16 of these 19 patients (84.2%), adding a second medication was sufficient, while 3 required a combination of three medications.

### Yield of the extended diagnostic work-up

Table 2 summarizes the results of the investigations conducted to define the etiology of hypertension in the cohort. The diagnostic yield of each test was calculated based on the percentage of contributory results relative to the total number of tests performed. Kidney US had the highest diagnostic yield (37.1%), revealing conditions such as congenital solitary kidney (*n* = 7), CAKUT-related CKD (*n* = 6), kidney hypo-dysplasia (*n* = 4), other CAKUT (*n* = 4), PKD (*n* = 3), and renal artery stenosis (*n* = 2). Advanced imaging techniques, such as CT angiography and MRI angiography, were performed only if the Doppler US of the renal arteries was abnormal, showing a percentage of contributory findings of 66.6% and 7.7%, respectively. Low-dose ACTH stimulation test and dexamethasone suppression test (DST) were performed when appropriate. DST contributed to diagnosing Cushing’s syndrome in a patient with Carney complex.

**Table 2:** Number of performed tests of the extended and short diagnostic work-up, their abnormal and contributory findings, and their costs. Tests included in the short diagnostic work-up are shown in italics

		Performed tests, *n*	Abnormal findings, *n*. (%)	Diagnostic criteria to define the abnormal findings	Contributory findings, *n*. (%)	Costs, €
First-line investigations	*Creatinine*	70	7 (10)	eGFR (mL/min/1.73 m^2^) < 90 for children aged > 2 years or according to specific reference values for children younger than 2 years of age: Preterm babies (1.5–4 months) < 34.2 Term babies (1–3 months) < 15.1 (0–3 months) < 25.6 (4–6 months) < 42.8 (7–12 months) < 71.8 (1–2 years) < 70.6	7 (10)	77.00
	*Glucose*	70	1 (1.4)	Fasting glucose > 100 mg/dL	1 (1.4)	70.00
	*Sodium*	70	1 (1.4)	< 135 mEq/L	1 (1.4)	59.50
	*Potassium*	70	3 (4.3)	< 3.5 mEq/L	3 (4.3)	70.00
	Chloride	70	0	< 98 mEq/L or > 107 mEq/L	0	63.00
	Lipid profile	70	11 (15.7)	Total cholesterol ≥ 200 mg/dL Triglycerides (0–9 years) ≥ 100 mg/dL (≥ 10 years) ≥ 130 mg/dL	0	150.50
	*Bicarbonate*	70	4 (5.7)	> 28 mEq/L	4 (5.7)	66.50
	Thyroid hormones	70	2 (2.8)	TSH (µUI/mL) (1–12 months) > 7 (1–6 years) > 6 (7–12 years) > 5 (> 12 years) > 4.5	0	360.50
	Plasma renin activity	70	12 (17.1)	(1–2 years) > 7.8 ng/h/mL (2–10 years) > 5.2 ng/h/mL (10–15 years) > 2.6 ng/h/mL (adult – upright 4 h) > 7.4 ng/h/mL (adult – supine 30 min) > 3.1 ng/h/mL	2 (2.8)	430.50
	Aldosterone	70	9 (12.8)	(1–2 years) > 54 ng/dL (2–10 years) > 29 ng/dL (10–15 years) > 22 ng/dL (adult – upright 4 h) > 30 ng/dL (adult – supine 30 min) > 16 ng/dL	2 (2.8)	546.00
	Blood cortisol	70	32 (45.7)	> 25 µg/dL (8:00 AM) > 14 µg/dL (24:00 PM)	1 (1.4)	311.50
	ACTH	70	11 (15.7)	< 7.2 or > 63 pg/mL (8:00 AM draws)	1 (1.4)	504.00
	DHEAS	70	0	(1–2 years) > 2.9 ng/mL (2–5 years) > 2.3 ng/mL (6–10 years) > 3.4 ng/mL (11–14 years) > 5.0 ng/mL (15–18 years) > 6.6 ng/mL	0	448.00
	17-OH progesterone	70	0	> 2 ng/mL	0	539.00
	Delta4-androstenedione	70	0	(Prepubertal) > 52 ng/dL (Adult) > 230 ng/dL	0	476.00
	*Urinalysis*	70	3 (4.3)	Presence of proteinuria or hematuria	3 (4.3)	178.50
	Urine ACR	70	4 (5.7)	≥ 30 mg/g	3^1^ (4.3)	322.00
	Beta2 microglobulin	70	5 (7.1)	> 0.3 mg/L	4^2^ (5.7)	227.50
	Urine metanephrines and normetanephrines	70	0	Metanephrines (3–8 years) > 240 µg/g creatinine (9–12 years) > 220 µg/g creatinine (13–18 years) > 145 µg/g creatinine Normetanephrines (3–8 years) > 705 µg/g creatinine (9–12 years) > 583 µg/g creatinine (13–18 years) > 375 µg/g creatinine	0	1386.00
	Urine adrenaline and noradrenaline	70	8 (11.4)	Adrenaline (< 2 years) > 75 µg/g creatinine (2–4 years) > 57 µg/g creatinine (5–9 years) > 35 µg/g creatinine (10–19 years) > 34 µg/g creatinine Noradrenaline (< 2 years) > 420 µg/g creatinine (2–4 years) > 120 µg/g creatinine (5–9 years) > 89 µg/g creatinine (10–19 years) > 82 µg/g creatinine	0^3^	1368.50
	Urine dopamine	70	15 (21.4)	(< 2 years) > 3000 µg/g creatinine (2–4 years) > 1533 µg/g creatinine (5–9 years) > 1048 µg/g creatinine (10–19 years) > 545 µg/g creatinine	0^4^	1090.60
	Urine vanillylmandelic acid	70	5 (7.1)	(1–4 years) > 16.2 mg/g creatinine (5–9 years) > 11.3 mg/g creatinine (10–19 years) > 9.1 mg/g creatinine	0^5^	1298.50
	Urine hydroxy-indoleacetic acid	70	0	(1–10 years) > 25 µg/g creatinine (10–18 years) > 30 µg/g creatinine	0	927.50
	Urine homovanillic acid	70	9 (12.8)	(1–4 years) > 22 mg/g creatinine (5–9 years) > 15.1 mg/g creatinine (10–19 years) > 12.8 mg/g creatinine	0^6^	948.50
	*Kidney US*	70	34 (48.6)	Solitary kidney Kidney hypo-dysplasia Other CAKUT Renal echo structure abnormalities such as cortical hyperechogenicity, altered corticomedullary differentiation, renal cysts, or reduction of parenchymal thickness	26 (37.1)	2646.00
	*Doppler US of the renal arteries*	70	15 (21.4)	Major criteria: Peak Systolic Velocity (PSV) > 180 cm/s Telediastolic velocity (VD) > 90 cm/s Minor criteria: Renal Artery Resistance Index (RI) > 0.7	2^7^ (2.8)	3150.00
						**Total costs of first-line investigations of the extended diagnostic work-up:** 17,715.60 **Total costs of the short diagnostic work-up = **6317.50
Second-line investigations	Computed tomography angiography	3	2 (66.6)	Arterial narrowing Delayed contrast washout or non-uniform contrast enhancement Post-stenotic dilation Collateral circulation	2^8^ (66.6)	424.35
	Magnetic resonance angiography	13	1 (7.7)	Arterial narrowing Delayed contrast washout Delayed enhancement Post-stenotic dilation Collateral circulation	1^8^ (7.7)	2402.40
	Low-dose ACTH stimulation test	3	0	Blood cortisol < 16 µg/dL	0	50.70
	DST	7	1 (14.3)	Blood cortisol > 1.8 µg/dL	1 (14.3)	118.30
						**Total costs of second-line investigations:** 2995.75

^1^In one patient (1.4%), we obtained abnormalities not confirmed in a subsequent dosage

^2^In one patient (1.4%), we obtained abnormalities not confirmed in a subsequent dosage

^3^In eight patients (11.4%), we obtained abnormalities not confirmed in a subsequent dosage

^4^In 15 patients (21.4%), we obtained abnormalities not confirmed in a subsequent dosage

^5^In five patients (7.1%), we obtained abnormalities not confirmed in a subsequent dosage

^6^In nine patients (12.8%), we obtained abnormalities not confirmed in a subsequent dosage

^7^In additional 13 patients, Doppler US of the renal arteries was pathological, but the subsequent computed tomography angiography or magnetic resonance angiography excluded renal artery stenosis

^8^One patient with renal artery stenosis underwent both computed tomography angiography and magnetic resonance angiography

*eGFR*, estimated glomerular filtration rate; *ACTH*, adrenocorticotropic hormone; *DHEAS*, dehydroepiandrosterone sulfate; *ACR*, albumin-creatinine ratio; *US*, ultrasound; *DST*, dexamethasone suppression test; *CAKUT*, congenital anomalies of the kidneys and of the urinary tract

### Yield of the short diagnostic work-up

**Fig. 2:**
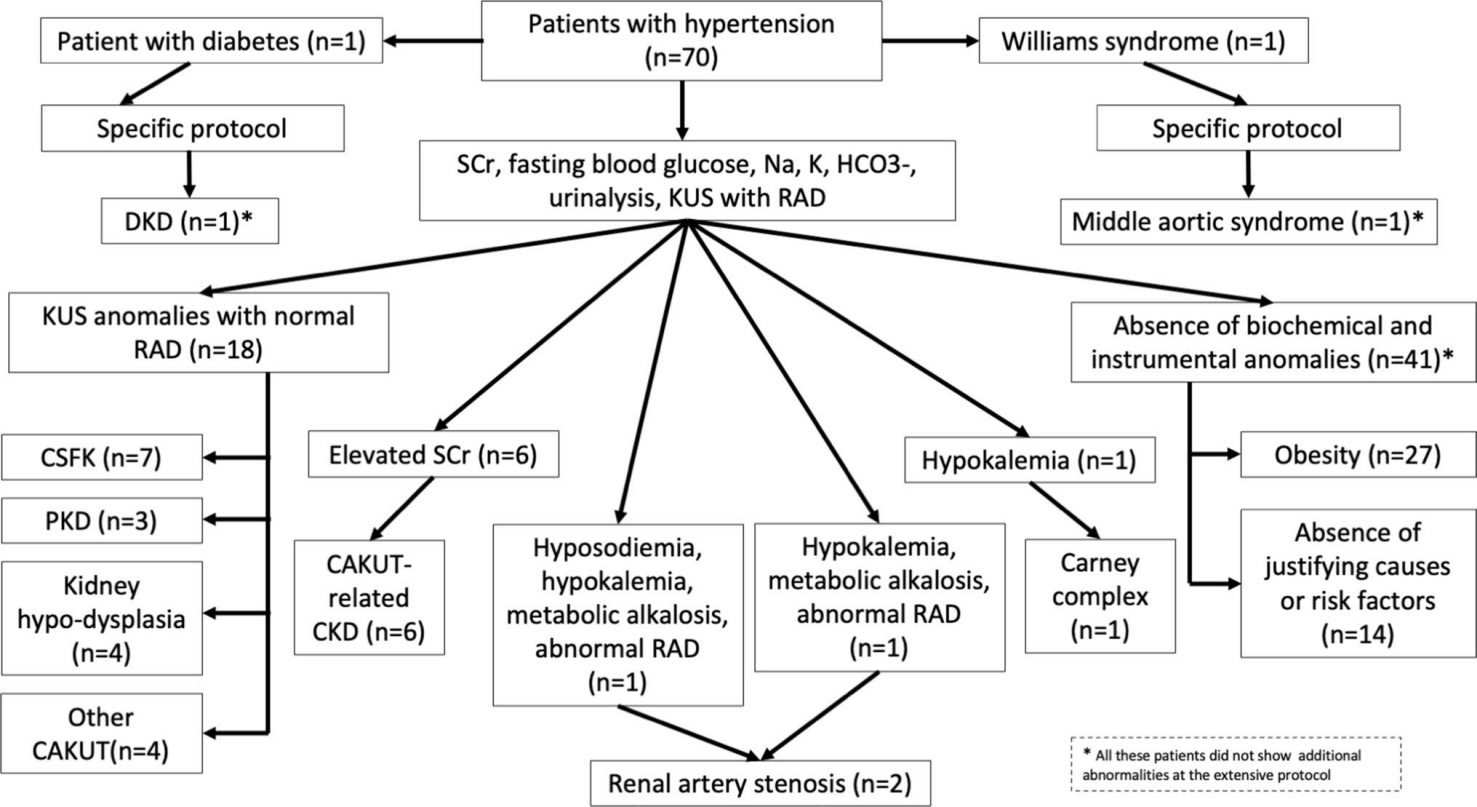
Performance of the short diagnostic work-up. SCr, serum creatinine; KUS, kidney ultrasound; RAD, Doppler ultrasound of the renal arteries; CSFK, congenital solitary functioning kidney; PKD, polycystic kidney disease; CAKUT, congenital anomalies of the kidney and urinary tract; CKD, chronic kidney disease; DKD, diabetic kidney disease

The performance of the short diagnostic work-up is shown in Fig. 2. The short diagnostic work-up successfully identified the cause or a suspected secondary form of hypertension in all 29 patients (100%) with secondary hypertension. Kidney US had the highest diagnostic yield, followed by serum creatinine and bicarbonates (Table 2).

### Costs analysis

The extensive first-line screening for the 70 enrolled patients incurred direct costs totaling € 17,715.60 (€ 253.08 for each patient) (Table 2). The total costs of second-line investigations were € 2995.75 (Table 2). Using the short first-line diagnostic work-up, the cost would have been € 6317.50 (€ 90.25 for each patient), representing a 64.3% cost reduction compared to the extensive protocol (Table 2).

### Comparison of the short diagnostic work-up with AAP and ESH guidelines

Table 3 compares the effectiveness and costs of our short diagnostic work-up with the testing protocols recommended by the AAP and ESH guidelines [1, 2]. The AAP protocol was less expensive than the short protocol (€ 19.08 vs. € 90.25 per patient), but it would have missed diagnoses of six cases of a congenital solitary kidney, two of PKD, three of kidney hypo-dysplasia, and four of other CAKUT. The AAP protocol resulted in a 92.4% cost savings compared to the extensive protocol. The ESH protocol was also less expensive than our short protocol (€ 60.30 vs. € 90.25 per patient) and would not have missed any secondary hypertension diagnoses in this cohort (Table 3). The ESH protocol achieved 76.2% cost savings compared to the extensive protocol.

**Table 3:** Number of performed tests and their costs comparing short diagnostic work-up, American Academy of Pediatrics protocol and European Society of Hypertension protocol

Short diagnostic work-up					American Academy of Pediatrics protocol					European Society of Hypertension protocol				
Investigations	Performed tests, *n*	Abnormal findings, *n*. (%)	Contributory findings, *n*. (%)	Costs, €	Investigations	Performed tests, *n*	Abnormal findings, *n*. (%)	Contributory findings, *n*. (%)	Costs, €	Investigations	Performed tests, *n*	Abnormal findings, *n*. (%)	Contributory findings, *n*. (%)	Costs, €
Creatinine	70	7 (10)	7 (10)	77.00	Creatinine	70	7 (10)	7 (10)	77.00	Creatinine	70	7 (10)	7 (10)	77.00
Glucose	70	1 (1.4)	1 (1.4)	70.00	Urea	70	0	0	70.00	Urea	70	0	0	70.00
Sodium	70	1 (1.4)	1 (1.4)	59.50	Sodium	70	1 (1.4)	1 (1.4)	59.50	Uric acid	70	0	0	73.50
Potassium	70	3 (4.3)	3 (4.3)	70.00	Potassium	70	3 (4.3)	3 (4.3)	70.00	Glucose	70	1 (1.4)	1 (1.4)	70.00
Bicarbonate	70	4 (5.7)	4 (5.7)	66.50	Chloride	70	0	0	63.00	Sodium	70	1 (1.4)	1 (1.4)	59.50
Urinalysis	70	3 (4.3)	3 (4.3)	178.50	Calcium	70	0	0	66.50	Potassium	70	3 (4.3)	3 (4.3)	70.00
Kidney US	70	34 (48.6)	26 (37.1)	2646.00	Phosphorus	70	0	0	70.00	Chloride	70	0	0	63.00
Doppler US of the renal arteries	70	15 (21.4)	2^1^ (2.8)	3150.00	Magnesium	70	0	0	77.00	Calcium	70	0	0	66.50
				Total costs = 6317.50	Lipid profile	70	11 (15.7)	0	150.50	Phosphorus	70	0	0	70.00
					Urinalysis	70	3 (4.3)	3 (4.3)	178.50	Magnesium	70	0	0	77.00
					Kidney US*	12	9 (75)	8 (66.6)	453.60	Lipid profile	70	11 (15.7)	0	150.50
									Total costs = 1335.60	Urinalysis	70	3 (4.3)	3 (4.3)	178.50
										Urine PCR	70	4 (5.7)	3^2^ (4.3)	227.50
										Urine ACR	70	4 (5.7)	3^3^ (4.3)	322.00
										Kidney US	70	34 (48.6)	26 (37.1)	2646.00
														Total costs = 4221.00

^*^If < 6 years old or if urinalysis or renal function is abnormal

^1^In additional 13 patients, Doppler US of the renal arteries was pathological, but the subsequent computed tomography angiography or magnetic resonance angiography excluded renal artery stenosis

^2^In one patient (1.4%), we obtained abnormalities not confirmed in a subsequent dosage

^3^In one patient (1.4%), we obtained abnormalities not confirmed in a subsequent dosage

*US*, ultrasound; *PCR*, protein-creatinine ratio; *ACR*, albumin: creatinine ratio

A cost-effective diagnostic approach, without missing any diagnoses, could be achieved by combining findings from the protocols. This approach involves the following first-line investigations to screen for secondary causes of hypertension: serum creatinine, fasting glucose, sodium, potassium, urinalysis, and kidney ultrasound (cost for each patient: € 44.30) (Table 4).

**Table 4:** First-line protocol resulting from the combination of our short protocol with the American Academy of Pediatrics and European Society of Hypertension protocols, along with their respective contributory findings and costs

Investigations	Performed tests, *n*	Abnormal findings, *n*. (%)	Contributory findings, *n*. (%)	Costs, €
Creatinine	70	7 (10)	7 (10)	77.00
Glucose	70	1 (1.4)	1 (1.4)	70.00
Sodium	70	1 (1.4)	1 (1.4)	59.50
Potassium	70	3 (4.3)	3 (4.3)	70.00
Urinalysis	70	3 (4.3)	3 (4.3)	178.50
Kidney US	70	34 (48.6)	26 (37.1)	2646.00
				Total costs = 3101.00

*US*, ultrasound

## Discussion

Traditionally, secondary hypertension has been considered the most common form of hypertension in childhood. However, recent trends indicate a shift in this paradigm [10–16]. In our retrospective study of 70 children with hypertension (mean age at presentation: 12.8 ± 4.7 years), 41.4% were found to have a secondary cause of hypertension. This finding aligns with recent literature, which indicates that essential hypertension is now the predominant form of pediatric hypertension, especially in patients over 6 years of age. This trend is largely attributed to the increasing prevalence of obesity and metabolic syndrome in children [10–16].

In fact, as age increased, the prevalence of secondary hypertension decreased (Supplementary Fig. 3). However, we want to highlight the high rate of secondary hypertension among 17-year-old patients. At this age, all cases of hypertension were related to CAKUT, likely because, with increasing age and after pubertal maturation, the congenital reduced nephron mass in patients with CAKUT becomes clinically evident, contributing to the development of hypertension.

Accurately identifying patients with secondary hypertension is crucial to effectively treating any underlying illnesses [1, 2]. Current guidelines recommend a case-by-case approach, considering factors such as age, medical history, clinical presentation, or treatment resistance [1, 2]. Interestingly, only 30 out of 70 patients in our cohort exhibited symptomatic hypertension, with headache being the most common symptom. Among these, only six symptomatic patients were found to have a secondary cause of hypertension, suggesting that the presence of symptoms alone is not a reliable indicator of secondary hypertension. Nevertheless, when symptoms are atypical or differ from headaches, the likelihood of secondary hypertension may increase.

To minimize diagnostic errors resulting from individualized and variable approaches, we implemented a standardized, comprehensive screening protocol in our clinical practice for all hypertensive patients. This approach aimed to ensure the identification of all secondary forms of hypertension. Notably, we also investigated potential secondary causes in patients whose characteristics, such as obesity, might typically suggest primary hypertension. Interestingly, some of these patients with obesity were ultimately diagnosed with secondary hypertension. This finding is consistent with the study of Kapur et al., which highlights that factors like obesity and the severity of hypertension should not preclude evaluation for secondary hypertension [17].

We analyzed our comprehensive screening for hypertensive children by differentiating between abnormal findings and contributory results and assessing the diagnostic utility of each test. Additionally, we conducted a cost analysis of the diagnostic work-up. Ding et al. [18] performed a similar study but without evaluating costs. They identified high-yield investigations, including kidney US, lipid profile for overweight or obese children, and echocardiograms to assess target organ damage. However, their study did not apply a uniform protocol of investigations to all participants [18]. As a result, the diagnostic yield was influenced by pre-test probabilities [18]. Similarly, Wiesen et al. [16] retrospectively evaluated the effectiveness of the protocol recommended by the Fourth Report [6] in patients with mild-to-moderate hypertension. Their findings indicated that routine urinalysis and serum biochemical tests were not particularly useful for diagnosing mild-to-moderate hypertension. The fasting lipid profile was the only blood test that consistently produced significant abnormal results. Additionally, they recommended the use of kidney US in cases with abnormal ABPM results or a strong clinical suspicion of renovascular hypertension or kidney parenchymal disease. However, this study had limitations including the lack of uniform examinations and the absence of a consistent, comprehensive diagnostic work-up.

In our cohort, kidney parenchymal or renovascular impairment emerged as the most common cause of secondary hypertension, consistent with findings from other studies [12, 13, 18]. Consequently, kidney US was the most contributory investigation in both the extended and short protocols. Abnormal creatinine levels were associated with CAKUT-related CKD. While urinalysis proved useful in evaluating hypertension, the ACR was less informative and primarily abnormal only in children with already abnormal urinalysis results. Urinalysis, however, demonstrates good sensitivity as a screening tool for macroalbuminuria (ACR > 300 mg/g), though its specificity is limited [19, 20]. Therefore, to detect also the cases of microalbuminuria (ACR between 30 and 299 mg/g), the ACR should be preferred [19, 20].

Hyponatremia was observed in one patient with renal artery stenosis, alongside hypokalemia, metabolic alkalosis, and abnormal findings on renal artery Doppler US. Similarly, the other patient in our cohort with renal artery stenosis exhibited hypokalemia, metabolic alkalosis, and abnormal Doppler US findings (Fig. 2). Doppler results were confirmed using CT/MRI angiographies, which also revealed abnormalities. The biochemical profiles of patients with renal artery stenosis in our study are consistent with the literature, as renal artery stenosis is known to be associated with metabolic alkalosis, hyperkalemia, and hyponatremia [21–23]. As described in the literature [24], hypokalemia was also identified in a patient with Cushing syndrome associated with the Carney complex, accompanied by abnormal cortisol, ACTH, and DST (Fig. 2). Abnormal lipid profiles were exclusively observed in children with overweight or obesity.

None of our patients showed elevated urinary levels of metanephrines, normetanephrines, adrenaline, noradrenaline, dopamine, vanillylmandelic acid, hydroxy-indoleacetic acid, or homovanillic acid that led to a diagnosis of pheochromocytoma as a secondary cause of hypertension. This is probably because pheochromocytoma or paraganglioma is more common in young adults, particularly between the ages of 30 and 40 [25]. However, we observed false-positive results in 28.6% of cases for catecholamines and in 12.8% for homovanillic acid.

It is crucial to emphasize that not all abnormal findings result in definitive diagnoses. Therefore, careful consideration is required to determine which patients should undergo further investigations. Universal testing may introduce confounding factors and create economic challenges.

While our extensive screening effectively diagnosed all secondary causes of hypertension, it incurred unjustifiable costs (€ 253.08 per patient) and produced a notable proportion of false-positive results. To address these limitations, we developed and tested a streamlined diagnostic protocol aimed at identifying all causes of secondary hypertension without missing diagnoses, while achieving a better cost–benefit ratio. We also compared this short diagnostic work-up with the guidelines of AAP and ESH (Table 3) [1, 2].

When comparing our extended and short diagnostic work-up, we found that the short protocol successfully identified the causes of hypertension or raised suspicion of a secondary hypertension in all patients identified by the extended protocol. Moreover, implementing the short diagnostic work-up resulted in a 64.3% cost saving in reaching the final diagnosis. The findings from the comparison of our short diagnostic work-up with the AAP and ESH guidelines [1, 2] are particularly noteworthy.

Both AAP and ESH protocols are less expensive than our short diagnostic work-up, with the AAP being the least costly (Table 3). However, the AAP protocol failed to identify several cases of secondary hypertension. This was primarily because it includes kidney US only for patients under 6 years old or for those with abnormal urinalysis or kidney function. In contrast, the ESH protocol, when applied to our cohort, successfully identified all cases of secondary hypertension. Both protocols included tests with a 0% rate of contributory findings, highlighting opportunities to further improve cost-effectiveness.

A detailed comparison between our short protocol and the ESH protocol [2] reveals that the main factor contributing to the cost difference is the routine use of Doppler US in our protocol. In the two patients with renal artery stenosis in our cohort, both had electrolyte abnormalities, and one exhibited kidney hypoplasia on US. As a result, the ESH protocol would have identified these patients as candidates for further investigation.

We acknowledge the limitations of Doppler US in diagnosing renal artery stenosis in children, including its susceptibility to false-negative and false-positive results. Nonetheless, we included Doppler US for all patients because it is a widely available, non-invasive imaging modality that avoids the risks associated with radiation and contrast agents [26]. Although its diagnostic accuracy is imperfect, Doppler US can identify patients with a higher suspicion of renal artery stenosis, guiding the need for confirmatory testing using more definitive modalities [26].

However, relying solely on Doppler US for diagnosis is insufficient. Based on the performance of the ESH protocol in our cohort, we propose that Doppler US should not be used routinely as a screening tool but reserved for cases with clinical suspicion of renal artery stenosis (e.g., electrolyte abnormalities, renal hypoplasia, poorly controlled hypertension).

Both AAP and ESH protocols also include tests such as urea, chloride, calcium, phosphorus, magnesium, and lipid profile [1, 2]. Additionally, the ESH protocol incorporates uric acid [2]. However, none of these tests showed contributory findings in our cohort and may not be strictly necessary. The ESH protocol also includes urine PCR and ACR [2], which in our cohort were abnormal only in patients with pre-existing abnormal urinalysis. These could potentially be omitted as routine screening tools, favoring urinalysis alone, as recommended by the AAP guidelines [1]. However, it is important to note that cases of isolated microalbuminuria (ACR between 30 and 299 mg/g) could be missed if only urinalysis is performed [19, 20].

We hypothesize that combining elements of our short diagnostic work-up with the AAP and ESH protocols could yield a cost-effective approach to screening for secondary causes of hypertension in children. This streamlined protocol would include serum creatinine, fasting glucose, sodium, potassium, urinalysis, and kidney US, with an estimated cost of € 44.30 per patient. Future studies and expert consensus are necessary before implementing this protocol in routine clinical practice (Table 4).

This study has several limitations, including its retrospective, single-center design, which requires validation in larger populations. However, single-center enrollment allowed for consistent clinical and radiological management, as well as standardized laboratory procedures. Furthermore, our center’s practice of extensive initial screening ensured that diagnostic yield was not influenced by the pre-test probabilities [18]. Selection bias is another limitation, as our university center specializes in managing children with CAKUT. Additionally, we often care for patients with complex endocrinopathies and diabetes, which necessitate hypertension evaluation. This likely accounts for the high rate of secondary causes observed in our study. Nevertheless, we believe this population reinforces the findings of our study by demonstrating the effectiveness of different protocols in screening for secondary hypertension in a high-risk cohort. However, our population is poorly representative of patients aged < 5 years, and additional data are needed in this age range. Finally, conditions such as pheochromocytoma or paraganglioma may be missed with the short protocol. Therefore, in cases presenting with typical symptoms (e.g., flushing and hypertensive peaks) or when hypertension is resistant to multiple drugs, a targeted diagnostic work-up should be initiated.

In conclusion, our study reinforces the recent trend indicating that primary hypertension is more prevalent than secondary hypertension in children, with kidney parenchymal disease being the most common cause of secondary hypertension. The short diagnostic work-up, costing € 90.25 per patient, successfully identified all cases of secondary hypertension detected by the extended protocol, achieving a 64.3% cost savings compared to the extended approach.

The most cost-effective strategy for screening secondary hypertension, however, appears to be the ESH protocol, with a cost of € 60.30 per patient (Table 3). By integrating elements of our short diagnostic work-up with the ESH and AAP protocols, focusing only on contributory tests, the cost could be further reduced to € 44.30 per patient without missing any diagnoses of secondary hypertension (Table 4).

When evaluating investigations in hypertensive children, it is crucial to emphasize that an abnormal result does not necessarily equate to a contributory finding. Thus, this study supports the AAP and ESH recommendations [1, 2], offering guidance on adopting a more cost-effective, simplified, and focused approach to managing children with elevated blood pressure. Additional testing can be tailored based on the context and the results of initial evaluations.

## Supplementary Information

Below is the link to the electronic supplementary material.

Supplementary Figure 1 CONSORT diagram showing patient enrollment

High resolution image (TIF 3068 KB)

Supplementary Figure 2 Distribution of hypertension and its causes by age. PKD, polycystic kidney disease; CAKUT, congenital anomalies of the kidney and urinary tract; CKD, chronic kidney disease; DKD, diabetic kidney disease

High resolution image (TIF 242 KB)

Supplementary Figure 3 Distribution of essential and secondary hypertension across age groups

High resolution image (TIF 138 KB)

Supplementary Figure 4 Clinical manifestations of hypertension

High resolution image (TIF 386 KB)

Graphical abstract (PPTX 145 KB)

## Data Availability

The datasets used and/or analyzed during the current study are available from the corresponding author on reasonable request.

## References

[1] Flynn JT, Kaelber DC, Baker-Smith CM (2017) Clinical practice guideline for screening and management of high blood pressure in children and adolescents. Pediatrics 140:e20171904 28827377 10.1542/peds.2017-1904

[2] Lurbe E, Agabiti-Rosei E, Cruickshank JK et al (2016) 2016 European Society of Hypertension guidelines for the management of high blood pressure in children and adolescents. J Hypertens 34:1887–1920. 10.1097/HJH.0000000000001039 27467768 10.1097/HJH.0000000000001039

[3] Italian Data Protection Authority (Garante per la protezione dei dati personali) (2014) Authorisation no. 9/2014-General Authorisation to Process Personal Data for Scientific Research Purposes [3786078]

[4] Flynn JT, Daniels SR, Hayman LL et al (2014) Cardiovascular risk in the pediatric population epidemiology of hypertension update: ambulatory blood pressure monitoring in children and adolescents A Scientific Statement From the American Heart Association AHA Scientific Statement. Hypertension 5:1116–1135. 10.1161/HYP.0000000000000007/-/DC1 10.1161/HYP.0000000000000007 PMC4146525 24591341

[5] Flynn JT, Urbina EM, Brady TM et al (2022) Ambulatory blood pressure monitoring in children and adolescents: 2022 update: A Scientific Statement from the American Heart Association. Hypertension 79:E114–E124. 10.1161/HYP.0000000000000215 35603599 10.1161/HYP.0000000000000215 PMC12168719

[6] National High Blood Pressure Education Program Working Group on High Blood Pressure in Children and Adolescents (2004) The fourth report on the diagnosis, evaluation, and treatment of high blood pressure in children and adolescents. Pediatrics 114(2):555–576 15286277

[7] (2023) Definizione delle tariffe dell’assistenza specialistica ambulatoriale e protesica. (23A04464). GU Serie Generale 181. https://www.gazzettaufficiale.it/atto/vediMenuHTML?atto.dataPubblicazioneGazzetta=2023-08-04&atto.codiceRedazionale=23A04464&tipoSerie=serie_generale&tipoVigenza=originario. Accessed 10 Aug 2024

[8] Lang RM, Badano LP, Victor MA et al (2015) Recommendations for cardiac chamber quantification by echocardiography in adults: an update from the American Society of Echocardiography and the European Association of Cardiovascular Imaging. J Am Soc Echocardiogr 28:1-39.e14. 10.1016/J.ECHO.2014.10.003 25559473 10.1016/j.echo.2014.10.003

[9] Wong TY, Mitchell P (2004) Hypertensive retinopathy. N Engl J Med 351:2310–2317. 10.1056/NEJMRA032865 15564546 10.1056/NEJMra032865

[10] Bassareo PP, Calcaterra G, Sabatino J et al (2023) Primary and secondary paediatric hypertension. J Cardiovasc Med 24:E77–E85. 10.2459/JCM.0000000000001432 10.2459/JCM.0000000000001432 37052224

[11] Avesani M, Calcaterra G, Sabatino J et al (2024) Pediatric hypertension: a condition that matters. Children 11:518. 10.3390/children11050518 38790513 10.3390/children11050518 PMC11120267

[12] Baracco R, Kapur G, Mattoo T et al (2012) Prediction of primary vs secondary hypertension in children. J Clin Hypertens 14:316–321. 10.1111/j.1751-7176.2012.00603.x 10.1111/j.1751-7176.2012.00603.x PMC8108809 22533658

[13] Çakıcı EK, Yazılıtaş F, Kurt-Sukur ED et al (2020) Clinical assessment of primary and secondary hypertension in children and adolescents. Arch Pediatr 27:286–291. 10.1016/j.arcped.2020.06.005 32682663 10.1016/j.arcped.2020.06.005

[14] De Simone G, Mancusi C, Hanssen H et al (2022) Hypertension in children and adolescents. Eur Heart J 43:3290–3301. 10.1093/eurheartj/ehac328 35896123 10.1093/eurheartj/ehac328

[15] Gupta-Malhotra M, Banker A, Shete S et al (2015) Essential hypertension vs. secondary hypertension among children. Am J Hypertens 28:73–80. 10.1093/ajh/hpu083 24842390 10.1093/ajh/hpu083 PMC4318949

[16] Wiesen J, Adkins M, Fortune S et al (2008) Evaluation of pediatric patients with mild-to-moderate hypertension: yield of diagnostic testing. Pediatrics 122:e988–e993. 10.1542/peds.2008-0365 18977966 10.1542/peds.2008-0365

[17] Kapur G, Ahmed M, Pan C et al (2010) Secondary hypertension in overweight and stage 1 hypertensive children: a Midwest Pediatric Nephrology Consortium report. J Clin Hypertens (Greenwich) 12:34–39. 10.1111/J.1751-7176.2009.00195.X 20047628 10.1111/j.1751-7176.2009.00195.x PMC8672976

[18] Ding FCL, Elias I, Wright R et al (2024) Yield of diagnostic testing in evaluating etiology and end organ effects of pediatric hypertension. Pediatr Nephrol 39:513–519. 10.1007/s00467-023-06101-x 37515741 10.1007/s00467-023-06101-x

[19] Collier G, Greenan MC, Brady JJ et al (2009) A study of the relationship between albuminuria, proteinuria and urinary reagent strips. Ann Clin Biochem 46:247–249. 10.1258/ACB.2009.008189 19264826 10.1258/acb.2009.008189

[20] Bökenkamp A (2020) Proteinuria—take a closer look! Pediatr Nephrol 35:533–541. 10.1007/S00467-019-04454-W/FIGURES/5 31925536 10.1007/s00467-019-04454-w PMC7056687

[21] Kurt-Sukur ED, Brennan E, Davis M et al (2022) Presentation, treatment, and outcome of renovascular hypertension below 2 years of age. Eur J Pediatr 181:3367–3375. 10.1007/S00431-022-04550-4 35792951 10.1007/s00431-022-04550-4 PMC9395438

[22] Ding JJ, Lin SH, Lai JY et al (2019) Unilateral renal artery stenosis presented with hyponatremic-hypertensive syndrome - case report and literature review. BMC Nephrol 20:64. 10.1186/S12882-019-1246-9 30791890 10.1186/s12882-019-1246-9 PMC6385391

[23] Kovalski Y, Cleper R, Krause I et al (2012) Hyponatremic hypertensive syndrome in pediatric patients: is it really so rare? Pediatr Nephrol 27:1037–1040. 10.1007/S00467-012-2123-Y 22366877 10.1007/s00467-012-2123-y

[24] Liu CH, Ge YL, Wu NJ, Jin XP (2019) Hyperglycemia and hypokalemia in a 16-year-old overweight female patient misdiagnosed with cushing syndrome at first and ultimately diagnosed with carney complex proven by PRKAR1A gene test: a case report and literature review. Clin Lab 65:411–413. 10.7754/CLIN.LAB.2018.180805 10.7754/Clin.Lab.2018.180805 30868845

[25] De Freminville JB, Gardini M, Cremer A et al (2024) Prevalence and risk factors for secondary hypertension in young adults. Hypertension 81:2340–2349 39297209 10.1161/HYPERTENSIONAHA.124.22753

[26] Chhadia S, Cohn RA, Vural G, Donaldson JS (2013) Renal Doppler evaluation in the child with hypertension: a reasonable screening discriminator? Pediatr Radiol 43:1549–1556. 10.1007/S00247-013-2741-Y 23860636 10.1007/s00247-013-2741-y

