# Supplementary figures and images for: Investigations for diagnosis of secondary hypertension in children: yield and costs
Source: Pediatr Nephrol. 2025 Mar 31;40(9):2919–32. doi: 10.1007/s00467-025-06716-2 (PMC12296987; doi:10.1007/s00467-025-06716-2)

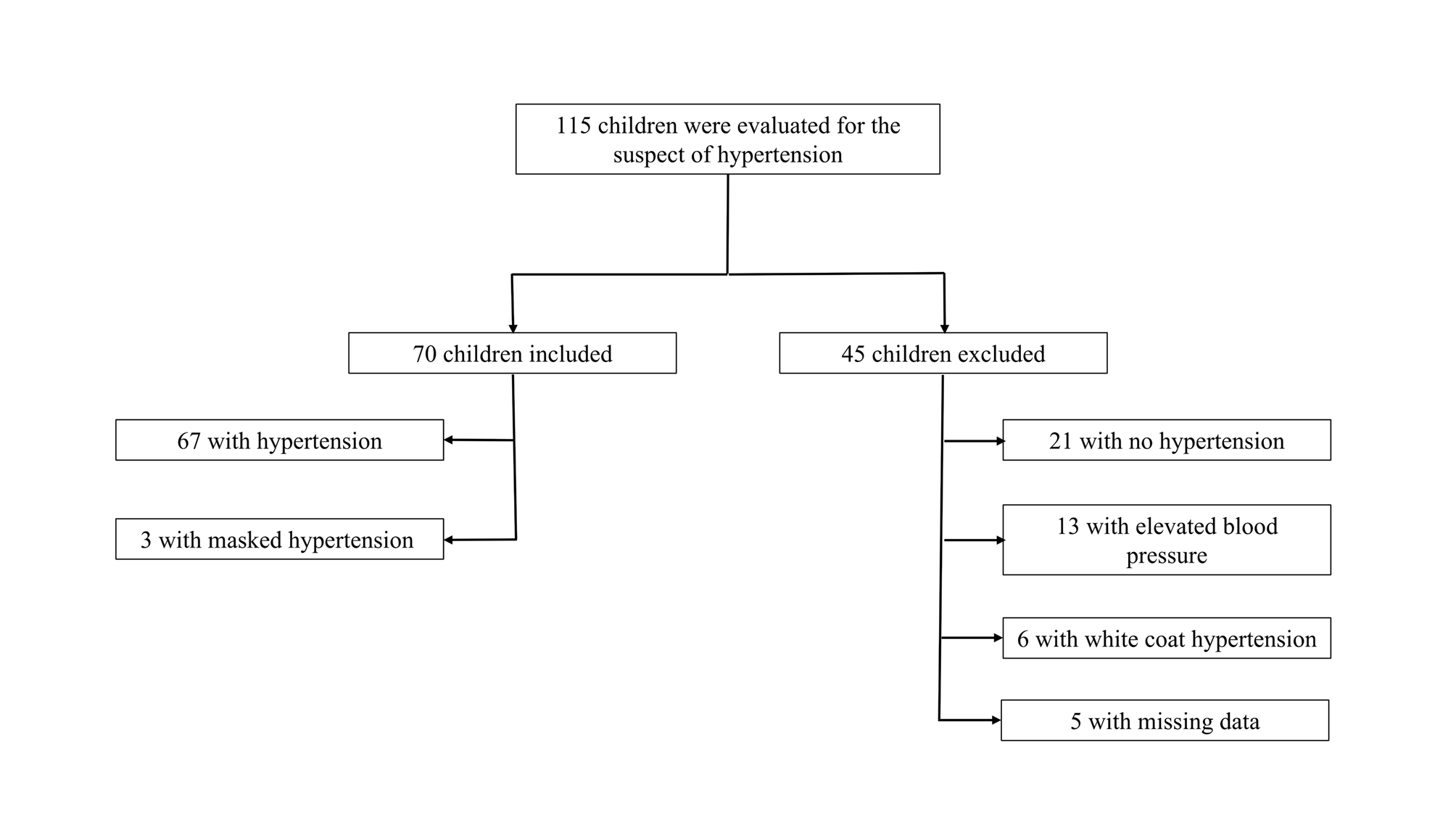

Supplement: Supplementary file 1 — CONSORT diagram showing patient enrollment [file 467_2025_6716_Fig3_ESM.png]

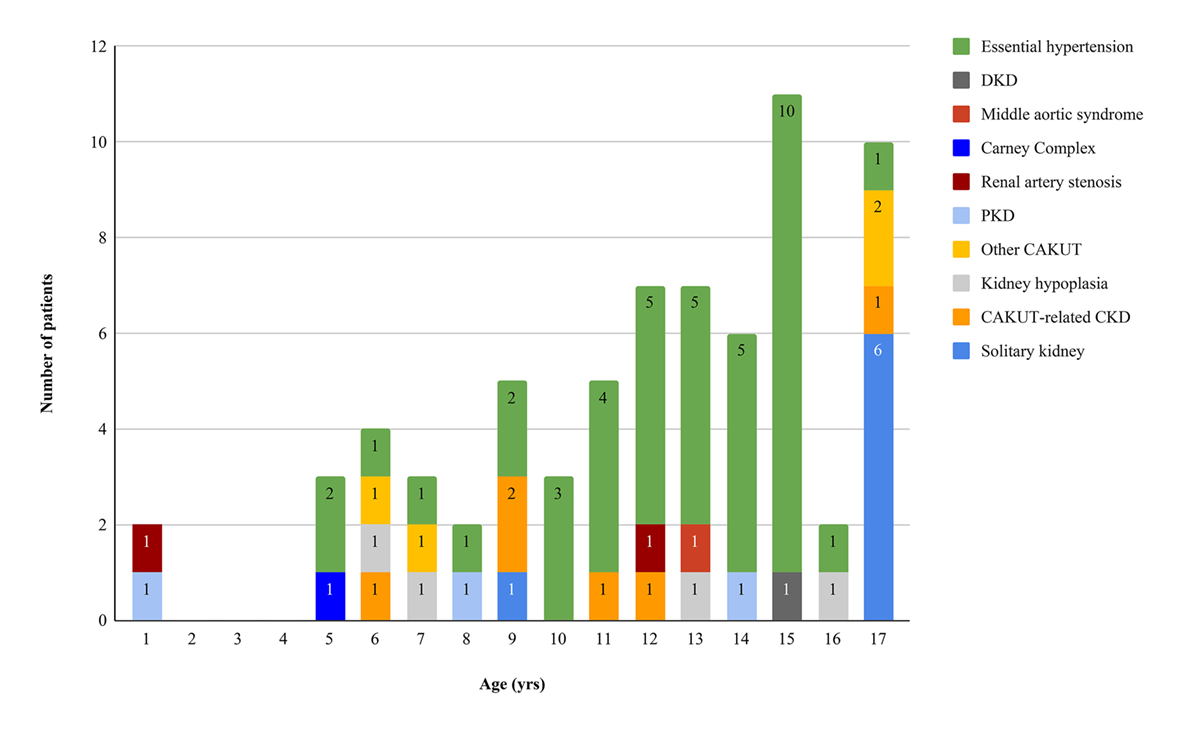

Supplement: Supplementary file 3 — Distribution of hypertension and its causes by age. PKD, polycystic kidney disease; CAKUT, congenital anomalies of the kidney and urinary tract; CKD, chronic kidney disease; DKD, diabetic kidney disease [file 467_2025_6716_Fig4_ESM.png]

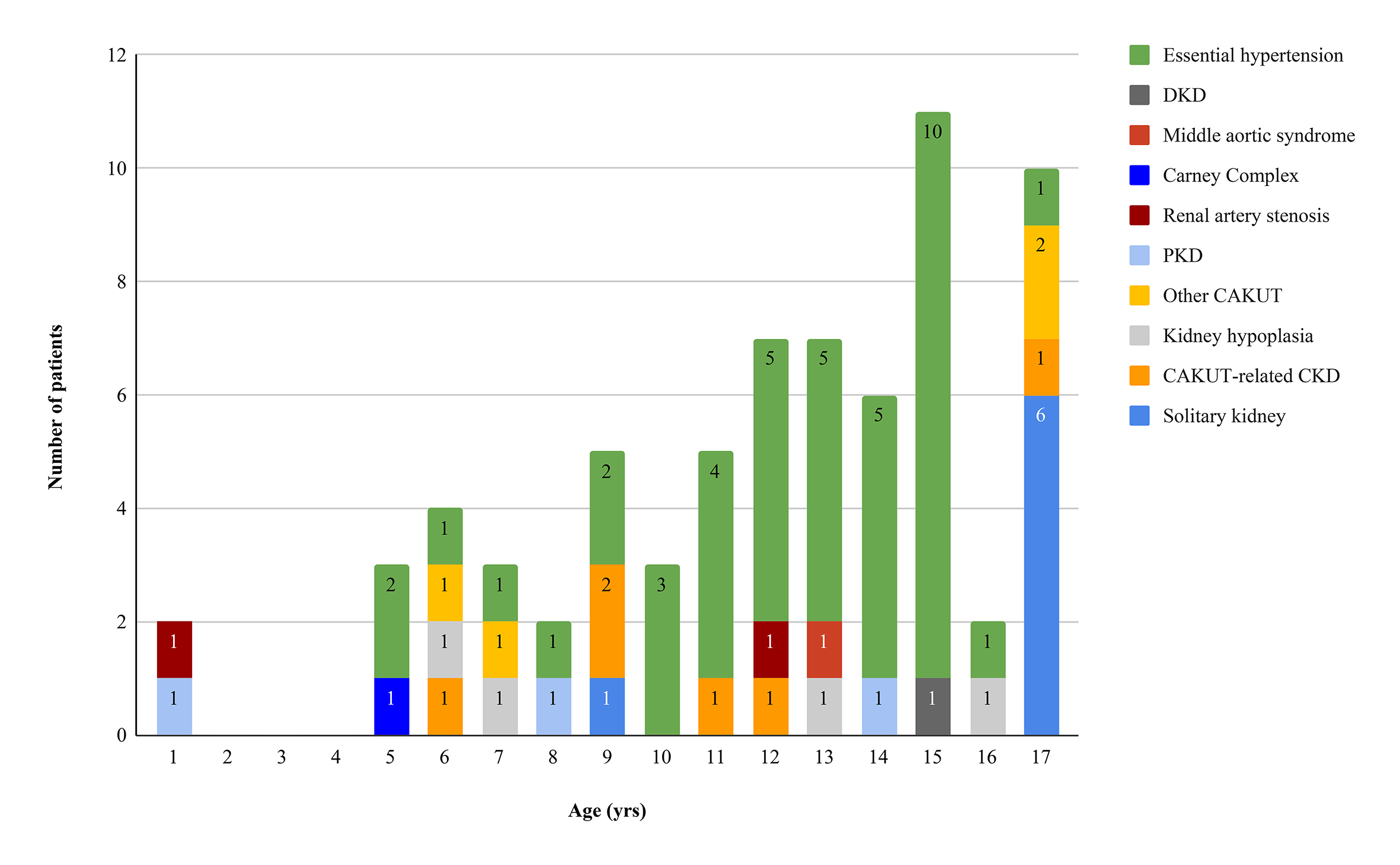

Supplement: Supplementary file 4 — High resolution image (TIF 242 KB) [file 467_2025_6716_MOESM2_ESM.tif]

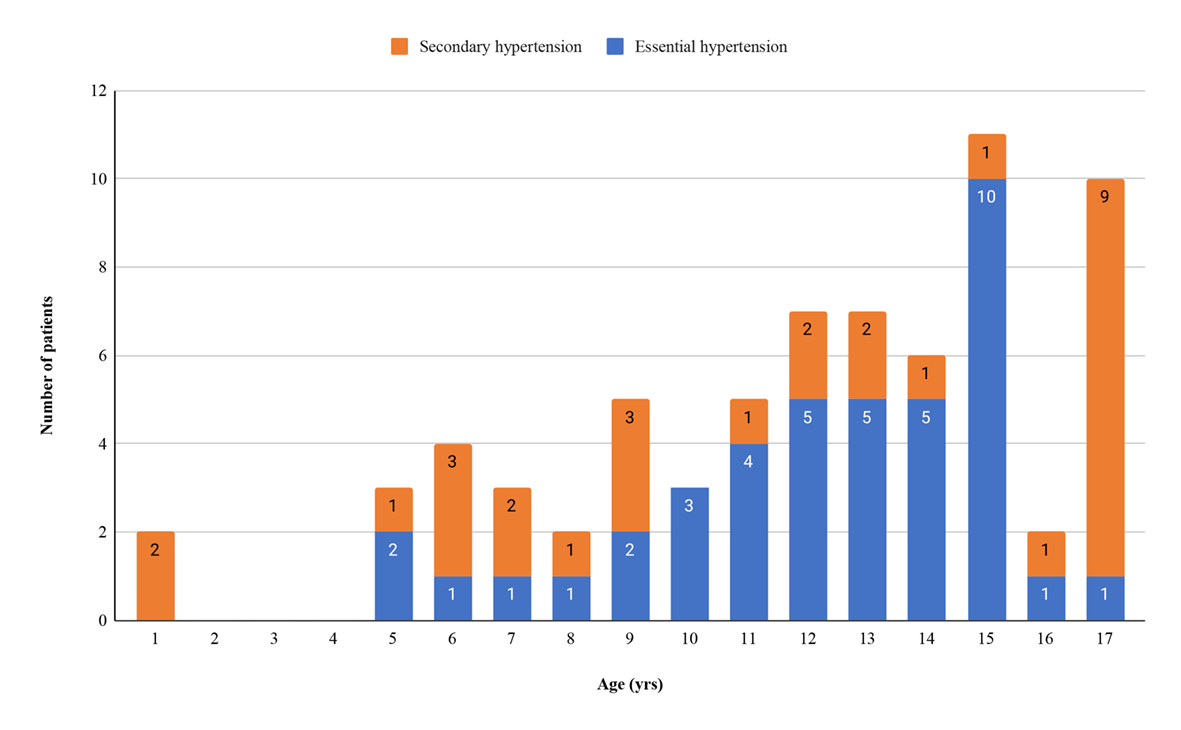

Supplement: Supplementary file 5 — Distribution of essential and secondary hypertension across age groups [file 467_2025_6716_Fig5_ESM.png]

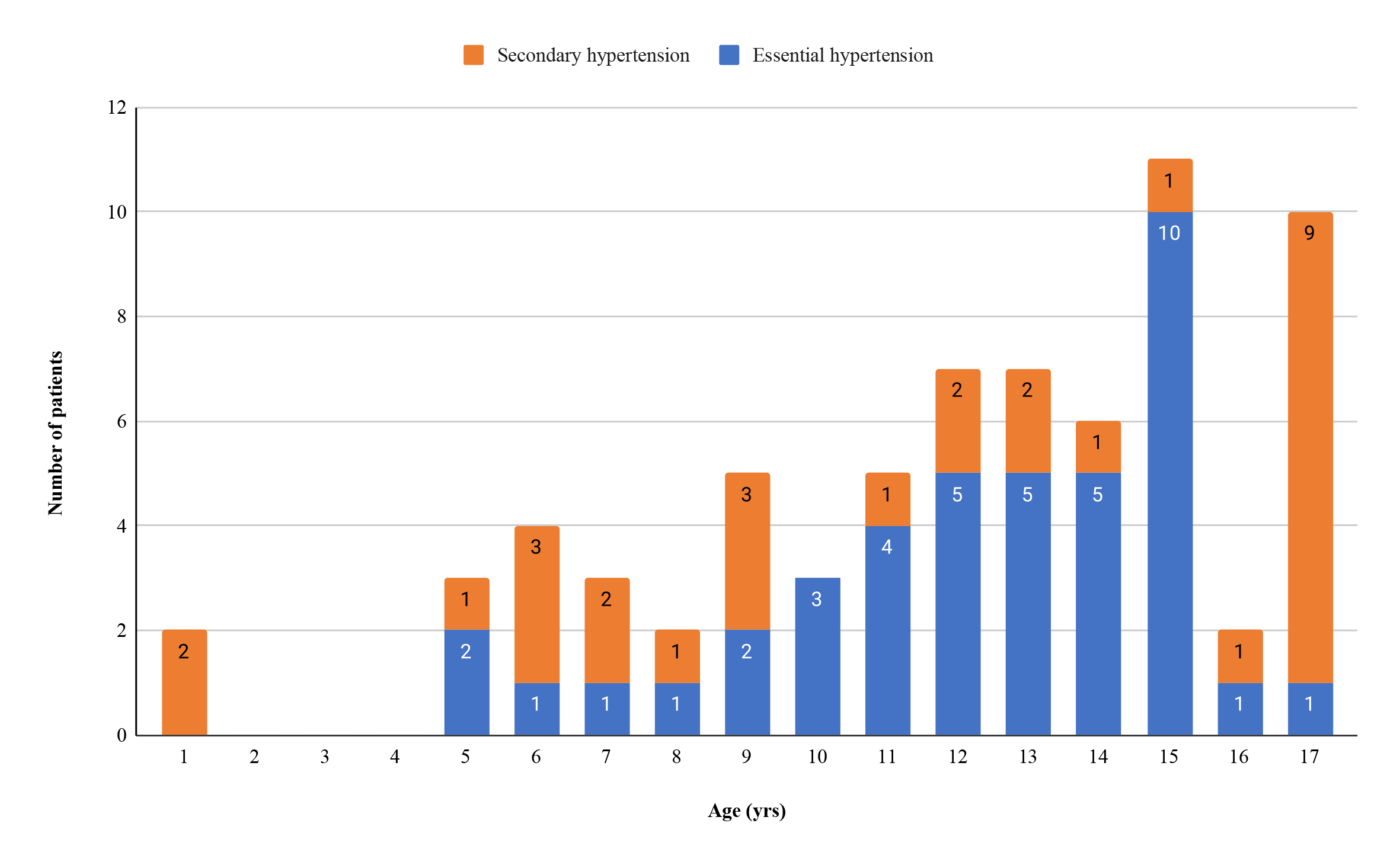

Supplement: Supplementary file 6 — High resolution image (TIF 138 KB) [file 467_2025_6716_MOESM3_ESM.tif]

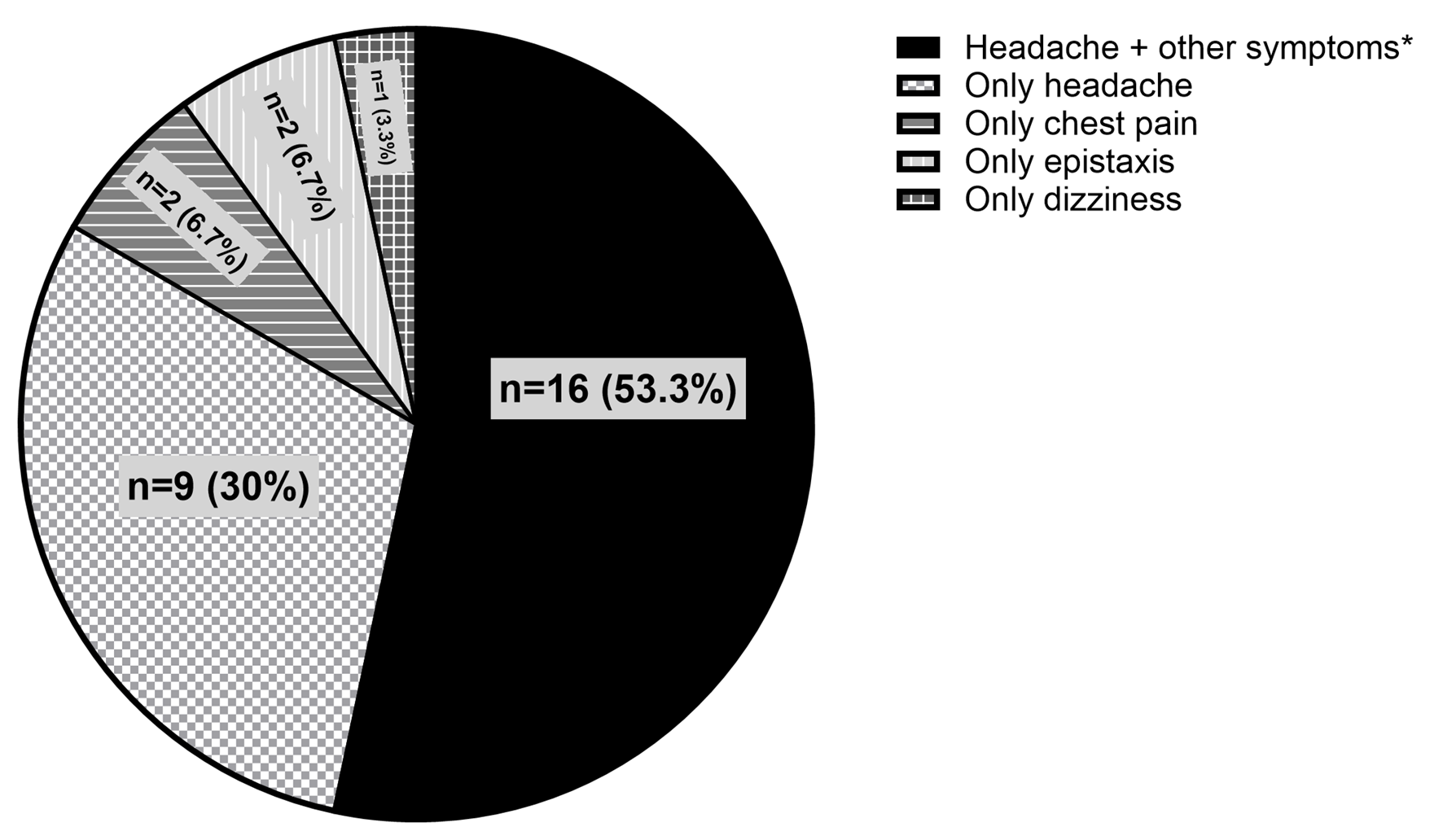

Supplement: Supplementary file 7 — Clinical manifestations of hypertension [file 467_2025_6716_Fig6_ESM.png]

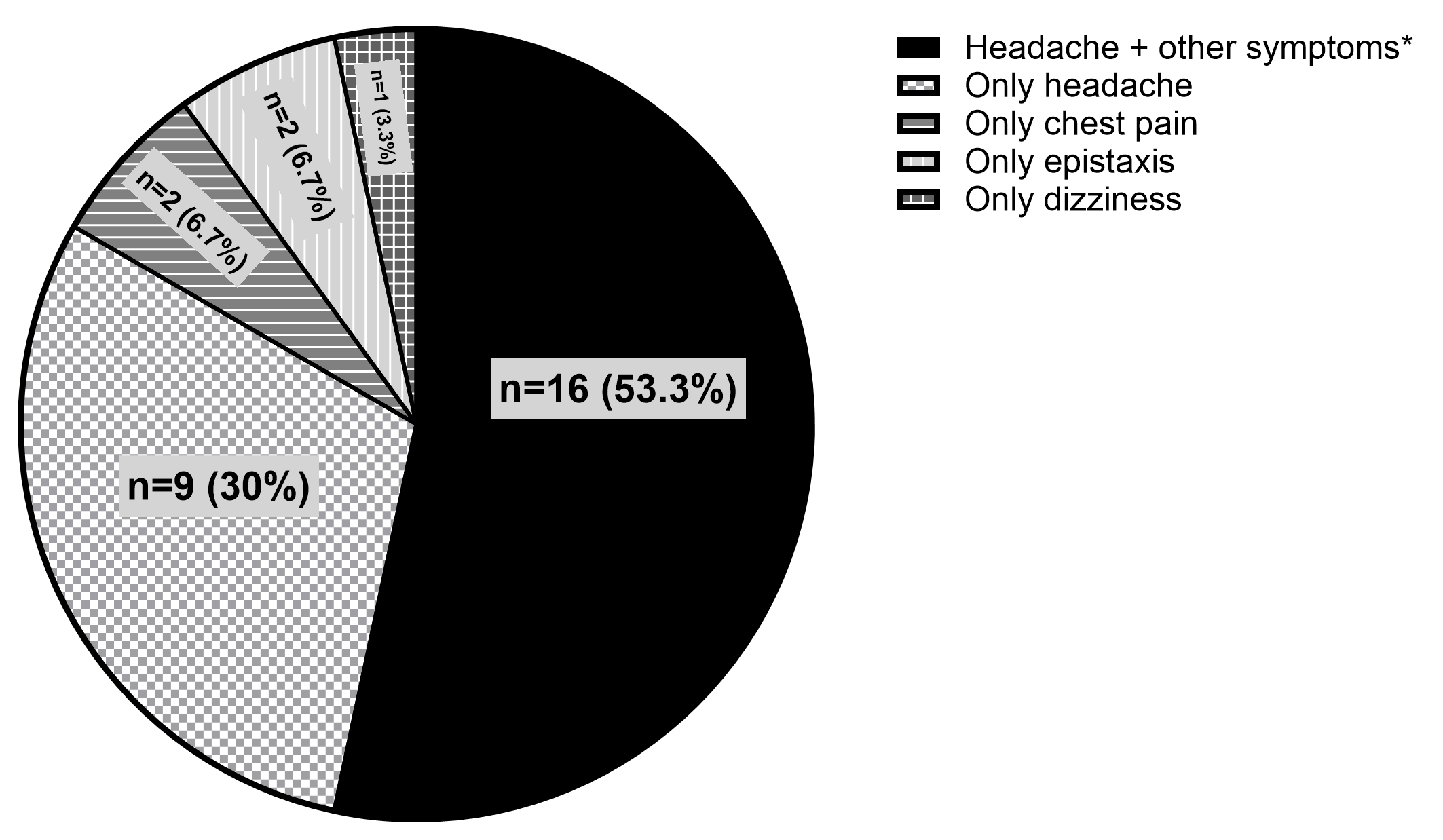

Supplement: Supplementary file 8 — High resolution image (TIF 386 KB) [file 467_2025_6716_MOESM4_ESM.tif]
