# Supplementary material for: Investigations for diagnosis of secondary hypertension in children: yield and costs
Source: Pediatr Nephrol. 2025 Mar 31;40(9):2919–32. doi: 10.1007/s00467-025-06716-2 (PMC12296987; doi:10.1007/s00467-025-06716-2)
Supplement: Supplementary file 9 — Graphical abstract (PPTX 145 KB) [file 467_2025_6716_MOESM5_ESM.pptx]

## Slide 1
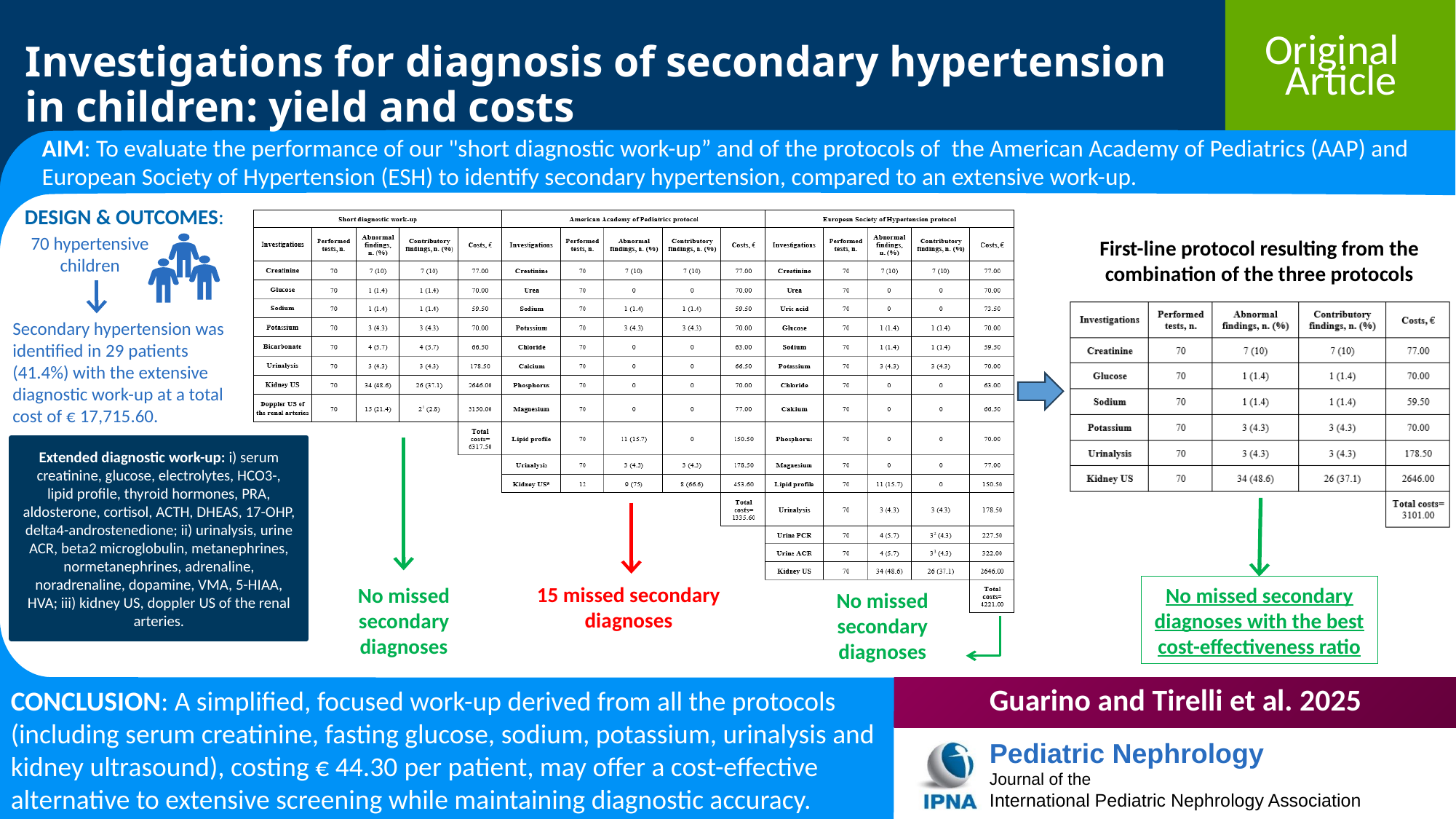

Investigations for diagnosis of secondary hypertension in children: yield and costs
AIM: To evaluate the performance of our "short diagnostic work-up” and of the protocols of the American Academy of Pediatrics (AAP) and European Society of Hypertension (ESH) to identify secondary hypertension, compared to an extensive work-up.
DESIGN & OUTCOMES:
70 hypertensive children
First-line protocol resulting from the combination of the three protocols
Secondary hypertension was identified in 29 patients (41.4%) with the extensive diagnostic work-up at a total cost of € 17,715.60.
Extended diagnostic work-up: i) serum creatinine, glucose, electrolytes, HCO3-, lipid profile, thyroid hormones, PRA, aldosterone, cortisol, ACTH, DHEAS, 17-OHP, delta4-androstenedione; ii) urinalysis, urine ACR, beta2 microglobulin, metanephrines, normetanephrines, adrenaline, noradrenaline, dopamine, VMA, 5-HIAA, HVA; iii) kidney US, doppler US of the renal arteries.
15 missed secondary diagnoses
No missed secondary diagnoses
No missed secondary diagnoses with the best cost-effectiveness ratio
No missed secondary diagnoses
Guarino and Tirelli et al. 2025
CONCLUSION: A simplified, focused work-up derived from all the protocols (including serum creatinine, fasting glucose, sodium, potassium, urinalysis and kidney ultrasound), costing € 44.30 per patient, may offer a cost-effective alternative to extensive screening while maintaining diagnostic accuracy.
